# Individual case safety reports in children in commonly used drug groups – signal detection

**DOI:** 10.1186/1472-6904-8-1

**Published:** 2008-03-17

**Authors:** Gertrud Brunlöf, Carina Tukukino, Susanna M Wallerstedt

**Affiliations:** 1 Department of Clinical Pharmacology and Regional Pharmacovigilance Centre, Sahlgrenska University Hospital, SE-413 45 Göteborg, Sweden

## Abstract

**Background:**

Due to few paediatric drug safety studies, knowledge on risks of drug treatment in children is limited. The knowledge needs to be increased to make proper risk-benefit analyses possible when treating paediatric patients with drugs. The aim of the present study was to investigate drug groups commonly used in children concerning type and frequency of individual case safety reports in children.

**Methods:**

Number and type of individual case safety reports in the 30 groups of drugs (5th level ATC-code) most sold (number of defined daily doses) in outpatient treatment to children (<15 years old) during 2005 were obtained. Descriptive analyses of the adverse drug reactions reported in children were performed.

**Results:**

The number of individual case safety reports per million defined daily doses in children varied in the groups of drug between 0 and 24. The largest number was found in the drug group R03DC, the leukotriene receptor antagonist montelukast; the majority of the children being <5 years old and experiencing psychiatric adverse drug reactions.

**Conclusion:**

The number of individual case safety reports per million defined daily doses varies in different groups of drugs. A possible signal for montelukast and psychiatric adverse drug reactions was found, which should be further explored.

## Background

Adverse drug reactions (ADRs) are a major health care problem. ADRs cause hospital care in both adults [1-3] and children [4]. Moreover, drug-related deaths have been reported for children [5,6]. Consequently, a risk-benefit analysis of drug treatment is essential in most patient consultations including paediatric patients. This implies access of adequate knowledge on both these parameters. Due to few paediatric drug safety studies, knowledge on risks in children is limited. At registration, little information on ADRs in children is available since many drugs have not been tested in children [7]. Off-label use of drugs in children results in questions to drug information centres [8] and has been reported to be extensive [9-11], reported to result in an increased risk of ADRs [9]. Risk-benefit analyses of drugs for children are therefore dependent on observations of ADRs and effects from clinical use.

Spontaneous reporting of ADRs is an important method for detection of signals, which is one aim of pharmacovigilance. An ADR signal is defined as a possible relationship between an adverse event and a drug, the relationship being unknown or incompletely documented previously. In Sweden, physicians, dentists and nurses are obliged to report (i) serious ADRs, (ii) ADRs not mentioned in the SPC, (iii) ADRs related to the use of new drugs (≤ 2 years on the market) except those labelled as common in the summary of product characteristics (SPC), and (iv) ADRs which incidence seems to increase. (MPA Code of Statutes, 2006 [12]). An individual case safety report (ICSR) can involve several ADRs. All ICSRs are reviewed and classified by trained nurses and physicians according to the WHO Collaborating Centre for International Drug Monitoring instructions concerning e.g. seriousness of the ADR before entered in the Swedish database for ADRs (SWEDIS). A serious ADR is defined as any untoward medical occurrence that at any dose: (i) results in death, (ii) requires inpatient hospitalisation or prolongation of existing hospitalisation, (iii) results in persistent or significant disability/incapacity or (iv) is life-threatening. A serious drawback of the spontaneous reporting system is that the number of ICSRs is small in proportion to incidence of ADRs [13,14].

The aim of the present study was to investigate drug groups commonly used in children concerning the type and frequency of ICSRs in children.

## Methods

Apoteket AB has monopoly of prescription drug sales in Sweden. A national prescription register (Xplain) was established in the late 1990s to improve possibilities for drug utilization studies. Data on age, sex and residential area of the patient, as well as information on the prescriber and the drug dispensed [e.g. number of defined daily doses (DDD) and costs] are routinely gathered when prescriptions are dispensed at Swedish pharmacies. In the present study, Xplain was used to obtain the 30 groups of drugs (5th level ATC-code) most sold [number of defined daily doses (DDDs)] in outpatient treatment to children (<15 years old) during January – December 2005 in Sweden. Within every group of drugs, ICSRs reported in children to SWEDIS during the same period were acquired. The number of ICSRs per million DDD in children was calculated. For comparison, the corresponding number of ICSRs per million DDD in adults (≥15 years old) was calculated. The ICSRs including ADRs in foetus/children to women who took the actual medicine were excluded. Vaccine reports were excluded, since no figures on DDD were available.

## Results

In 19 of the 30 most sold groups of drugs in children, at least one ICSR was found (table 1). Totally 60 ICSRs were found. The number of ICSRs per million DDD in children varied in the groups of drug between 0 and 24. The largest number was found in the drug group R03DC, leukotriene receptor antagonists, the ADRs being described in table 2.

**Table 1:** ICSRs in children (<15 years old) and adults (≥15 years old) during 2005.

		**Children**				**Adults**			
**ATC code**	**Name of group**	**ICSR (n)**	**Serious ADR (n)**	**Million DDD (n)**	**ICSR per million DDD (95% CI)**	**ICSR (n)**	**Serious ADR (n)**	**Million DDD (n)**	**ICSR per million DDD (95% CI)**

R03DC	Leukotriene receptor antagonists	16	0	0.7	24 (13.6 – 38.6)	7	0	5.2	1.4 (0.5 – 2.8)
N06BA	Centrally acting sympathomimetics	12	0	1.5	7.8 (4.0 – 13.6)	15	2	2.6	5.7 (3.2 – 9.5)
H02AB	Glucocorticoids	3	0	0.7	4.5 (0.9 – 13.0)	31	21	32	1.0 (0.7 – 1.4)
H01BA	Vasopressin and analogues	3	0	1.1	2.8 (0.6 – 8.2)	5	2	1.3	3.9 (1.3 – 9.2)
N03AX	Other antiepileptics	1	0	0.4	2.4 (0.1 – 13.1)	58	19	9.7	6.0 (4.5 – 7.7)
R03CC	Selective beta-2-adrenoreceptor agonists	1	0	0.4	2.3 (0.1 – 12.6)	1	1	1.4	0.7 (0.02–3.9)
J01CA	Penicillins with extended spectrum	1	0	0.5	2.1 (0.1 – 11.5)	12	3	3.9	3.1 (1.6 – 5.4)
R05FA	Opium derivatives and expectorants	1	1	0.5	2.0 (0.1 – 11.4)	1	0	11	0.1 (0.002 – 0.5)
J01CE	Beta-lactamase sensitive penicillins	3	0	1.6	1.8 (0.4 – 5.3)	14	5	11	1.3 (0.7 – 2.1)
A10AE	Insulins and analogues. long-acting	1	0	0.7	1.5 (0.04–8.5)	7	2	11	0.6 (0.3 – 1.3)
R06AX	Other antihistamines for systemic use	5	1	3.4	1.5 (0.5 – 3.5)	14	3	31	0.5 (0.3 – 0.8)
H01AC	Somatropin and somatropin agonists	1	0	0.8	1.3 (0.03–7.1)	2	0	0.8	2.6 (0.3 – 9.5)
R03BA	Glucocorticoids, inhalants	4	0	3.6	1.1 (0.3 – 2.8)	5	0	37	0.1 (0.04 – 0.3)
R03AK	Adrenergics and other drugs for obstructive airway diseases	2	0	2.0	1.0 (0.1 – 3.7)	10	1	33	0.3 (0.1 – 0.6)
R05CB	Mucolytics	1	0	1.2	0.8 (0.02–4.5)	3	1	28	0.1 (0.02 – 0.3)
R06AE	Piperazine derivatives	1	0	1.5	0.7 (0.02–3.8)	3	1	19	0.2 (0.03 – 0.5)
R03AC	Selective beta-2-adrenoreceptor agonists	2	1	4.4	0.5 (0.1 – 1.7)	5	0	49	0.1 (0.03 – 0.2)
D07AA	Corticosteroids. weak (group I)	1	0	4.9	0.2 (0.01 – 1.1)	0	0	6.3	0.0 (-0.6)^1^
D02AX	Other emollients and protectives	1	0	53	0.02 (0.001 – 0.1)	0	0	149	0 (-0.02)^1^

ADR, adverse drug reaction; CI, confidence interval; DDD, defined daily dose; ICSR, individual case safety report

^1^one-sided 97.5% CI

**Table 2:** Description of ICSRs for children in the ATC code R03DC. All ICSRs concerned the substance montelukast.

**Age (years)**	**Dose (mg/day)**	**Treatment duration (when known)**	**ADR**	**SPC (Yes/No)**
4	4	-	Night mares	Yes
3	4	-	Sleep disorders	Yes
3	4	-	Cranial nerve lesion	No
4	4	2 years	Haemorrhage	Yes
			Pruritus	Yes
			Abdominal pain	Yes
			Rectal pain	No
2	4	-	Fever	No
			Fatigue	Yes
			Rash	No
1	4	4 days	Anxiety	No
3	4	-	Night mares	Yes
			Aggressiveness	Yes
2	4	4 days	Aggressiveness	Yes
3	4	continuing	Night mares	Yes
3	4	2 doses	Asthma aggravated	No
2	4	11 days	Rash	No
			Pruritus	Yes
			Sleep disorder	Yes
			Night mares	Yes
1	4	2 weeks	Anxiety	No
			Sleep disorder	Yes
6	5	8 weeks	Xerophthalmia	No
5	4	3 weeks	Appetite increased	No
8	5 (on demand)	5 weeks	Leucopenia	No
			Red blood cell disorder	No
14	10	2 years	Arthralgia	Yes
			Myalgia	Yes

ADR, adverse drug reaction; ICSR, individual case safety report; SPC, summary of product characteristics

The second largest number of ICSRs was found in the drug group centrally acting sympathomimetics. These reports concerned children 7 – 12 years old, experiencing ADRs during treatment with methylphenidate (n = 10) and atomoxetine (n = 2). ADRs reported more than once were gastro-intestinal (n = 6), skin (n = 4), body as a whole – general (n = 4), neurologic (n = 3), psychiatric (n = 3) and cardiovascular (n = 3) disorders, according to the WHO adverse reaction terminology preferred term. None of the ICSRs were classified as serious.

Three ICSRs were classified as serious ADRs according to the WHO definition. These reports concerned R05FA Opium derivatives and expectorants [ethylmorhpine (pancreatitis)], R06AX Other antihistamines for systemic use [desloratadine (fatigue, xerostomia, nausea, abdominal pain)] and R03AC Selective beta-2-adrenoreceptor agonists [terbutaline (vomiting)].

## Discussion

ICSRs were present in 19 of the 30 most commonly used drug groups in children. The number of ICSRs varied between the groups of drugs, the two most reported drug groups being the leukotriene receptor antagonists and centrally acting sympathomimetics. The reporting of new drugs should be expected to be larger compared with old drugs, according to the Swedish instructions concerning ADR reporting. The leukotriene receptor antagonist montelukast was registered in 1998. Consequently, no extra attention to ADRs during montelukast treatment was demanded in 2005. Centrally acting sympathomimetics, on the other hand, were introduced later and the number of ICSRs may be influenced by the increased focus on this drug group. Another explanation for increased reporting rates for certain drug groups may be media attention.

ADRs during treatment with montelukast seem to occur predominantly in small children, the majority in the present study being <5 years old. In the SPC of montelukast, nightmares and sleep disorders as well as aggressiveness are labelled as scarce ADRs. Anxiety, the diagnosis in two ICSRs in the present study, is not labelled in the SPC. The number of paediatric patients being reported to experience these psychiatric symptoms in the present study is quite large and may thus be a signal, worthwhile to explore further. Additional studies are needed to confirm or contradict the signal.

The ADRs reported during treatment with sympathomimetics were generally labelled in the SPC, thus known previously. The only reported ADR not specified in the SPC was an obsessive reaction, whereas anxiety in general is mentioned in the SPC.

In hospitalized children, the overall incidence of ADRs has been reported to be 9.5% and in outpatient patients the corresponding figure was 1.5% [4]. Hence, the number of ICSR in the present study indicates that there is an under-reporting of ADRs not only in adults, as previously shown [13,14], but also in children. The frequency of under-reporting in children needs to be further explored. Furthermore, the present study only allows conclusions concerning the paediatric population <15 years old, whereas children according to European Medicines Agency include 0 to 17 years.

In the present study, five percent of the ICSRs in children included serious ADRs. The corresponding figure for adults was 32%. With vaccine reports included, the proportion of serious ADRs has been reported to be 13% in children [4].

The design of the present study does not to allow conclusions concerning the question whether the number of ICSRs per million DDD differs between children and adults. Lower doses are often used in children, making direct comparisons difficult. Moreover, dose adjustments for children compared with DDD may vary depending on age of the child as well as the drug in question, making comparisons using DDD as denominator inconclusive. The number of ICSRs in the present study is quite small, implying that minor fluctuations in the number of reports can significantly affect the result. Hence, the disposition of ADRs in children needs further investigation.

## Conclusion

In conclusion, the present study indicates that ADRs are reported for commonly used drugs in children. The number of ICSRs varies in different groups of drugs. A possible signal for montelukast and psychiatric adverse drug reactions was found, which should be further explored.

## Competing interests

The author(s) declare that they have no competing interests.

## Authors' contributions

GB and CT participated in the design of the study, carried out the acquisition of data and revised the manuscript. SW conceived the study, participated in its design and drafted the manuscript. All authors read and approved the final manuscript.

## Pre-publication history

The pre-publication history for this paper can be accessed here:



## References

[B1] Mjörndal T, Boman MD, Hägg S, Backstrom M, Wiholm BE, Wahlin A, Dahlqvist R (2002). Adverse drug reactions as a cause for admissions to a department of internal medicine. Pharmacoepidemiol Drug Saf.

[B2] Schneeweiss S, Hasford J, Gottler M, Hoffmann A, Riethling AK, Avorn J (2002). Admissions caused by adverse drug events to internal medicine and emergency departments in hospitals: a longitudinal population-based study. Eur J Clin Pharmacol.

[B3] van den Bemt PM, Egberts AC, Lenderink AW, Verzijl JM, Simons KA, van der Pol WS, Leufkens HF (1999). Adverse drug events in hospitalized patients. A comparison of doctors, nurses and patients as sources of reports. Eur J Clin Pharmacol.

[B4] Impicciatore P, Choonara I, Clarkson A, Provasi D, Pandolfini C, Bonati M (2001). Incidence of adverse drug reactions in paediatric in/out-patients: a systematic review and meta-analysis of prospective studies. Br J Clin Pharmacol.

[B5] Kimland E, Rane A, Ufer M, Panagiotidis G (2005). Paediatric adverse drug reactions reported in Sweden from 1987 to 2001. Pharmacoepidemiol Drug Saf.

[B6] Moore TJ, Weiss SR, Kaplan S, Blaisdell CJ (2002). Reported adverse drug events in infants and children under 2 years of age. Pediatrics.

[B7] Impicciatore P, Choonara I (1999). Status of new medicines approved by the European Medicines Evaluation Agency regarding paediatric use. Br J Clin Pharmacol.

[B8] Kimland E, Bergman U, Lindemalm S, Bottiger Y (2006). Drug related problems and off-label drug treatment in children as seen at a drug information centre. Eur J Pediatr.

[B9] Horen B, Montastruc JL, Lapeyre-Mestre M (2002). Adverse drug reactions and off-label drug use in paediatric outpatients. Br J Clin Pharmacol.

[B10] Ufer M, Kimland E, Bergman U (2004). Adverse drug reactions and off-label prescribing for paediatric outpatients: a one-year survey of spontaneous reports in Sweden. Pharmacoepidemiol Drug Saf.

[B11] Turner S, Nunn AJ, Fielding K, Choonara I (1999). Adverse drug reactions to unlicensed and off-label drugs on paediatric wards: a prospective study. Acta Paediatr.

[B12] (2006). Medical Products Agency's Code of Statutes. LVFS.

[B13] Alvarez-Requejo A, Carvajal A, Begaud B, Moride Y, Vega T, Arias LH (1998). Under-reporting of adverse drug reactions. Estimate based on a spontaneous reporting scheme and a sentinel system. Eur J Clin Pharmacol.

[B14] Bäckström M, Mjörndal T, Dahlqvist R (2004). Under-reporting of serious adverse drug reactions in Sweden. Pharmacoepidemiol Drug Saf.

